# A new *Zanclognatha* from eastern North America and a preliminary key to the larvae of the genus (Lepidoptera, Erebidae, Herminiinae)

**DOI:** 10.3897/zookeys.149.2348

**Published:** 2011-11-24

**Authors:** David L. Wagner, Timothy L. McCabe

**Affiliations:** 1 Department of Ecology and Evolutionary Biology, University of Connecticut, Storrs, Connecticut 06269; 2 New York State Museum, Albany, New York, 12230

**Keywords:** detritus feeding, Herminiinae, larvae, *Zanclognatha*, species radiation

## Abstract

The adult of a widespread but previously undescribed species of *Zanclognatha* Lederer is described from eastern North America. Images of the mature larva and life history datafor *Zanclognatha* *dentata* **sp. n.** are included, along with a preliminary key to the larvae of ten eastern North American *Zanclognatha* species.

## Introduction

More than a dozen species of *Zanclognatha* Lederer, 1857 occur in eastern North America, often with six or more species flying at a single location. Alpha diversity and abundance of *Zanclognatha* tend to be highest in mixed hardwood and conifer woodlands and forests with an accumulation of leaf litter. The Nearctic *Zanclognatha* fauna bears earmarks of a recent radiation: several species are weakly differentiated or confused(e.g., *Zanclognatha gypsalis*-*minoralis*-*theralis* complex; genitalic diversity across the genus is modest in both sexes; and some species-level taxa occur mainly north of recent glacial maxima, e.g., *Zanclognatha lutalba* (Smith, 1906). In addition to the new species described here, which has long been confused with *Zanclognatha protumnusalis* (Walker, 1859), additional eastern *Zanclognatha* await recognition, e.g., the undescribed species near *Zanclognatha lituralis* (Hübner, 1818) mentioned by Rings et al. (1992).

The most recent revisionary study of *Zanclognatha* and its relatives is that of Owada (1987) based on his studies of the diverse Japanese herminiine fauna. In addition to *Zanclognatha*, his treatment includes all allied genera. Owada recognized both *Zanclognatha* and *Polypogon* Schrank (1802) as valid, with the former genus defined by having the labial palpus sickle-shaped and upcurved over the vertex; male antenna with a knot; M2, M3, and CuA1 not stalked in the forewing; hindwing with cell extending to nearly ½ and M2 arising from above the anal angle (of cell); male foreleg with tibial sheath; and male foretarsus five-segmented with first segment lacking a projection. *Zanclognatha dentata*sp. n., sharing the above features, falls unambiguously within Owada’s concept of the genus, despite the fact that the male genitalia of *Zanclognatha dentata* and other Nearctic members of the genus *Zanclognatha*, superficially resemble those of *Polypogon tentacularia* (L.), the type of *Polypogon*, in possessing a deeply emarginate valve with two primary lobes. The type-species of *Zanclognatha*, *Zanclognatha lunalis* Scopoli, and its Palearctic relatives, have a valve that is comprised of three lobes. Starting with Smith (1895), Lederer’s *Zanclognatha* has been used for the Nearctic herminiines with an antennal knot and upcurved labial palpus. Poole (1989) transferred North American *Zanclognatha* into *Polypogon*, but later, moved all species back into *Zanclognatha* (Poole and Lewis 1996).

The new species was “discovered” while reviewing larval images of *Zanclognatha protumnusalis* and *Zanclognatha martha* Barnes, 1928. Larval images indicated that there were three species involved (see Figs 8–11 and key). Below we describe the adult, provide images of the last instar, and discuss the biology of *Zanclognatha dentata* sp. n. We also include a key to the larvae of ten species of *Zanclognatha* found in eastern North American.

## Methods and materials

A cohort of larvae was reared from a female collected by Dale F. Schweitzer from New Jersey, Atlanta County: Egg Harbor Township, Absecon Creek on 15 July 2002, DLW Lot Number 2002G117. Larvae were reared on dead, browned, lightly moistened oak leaves. Surface as well as partially decayed leaves were provided (see Hohn and Wagner 2000). Specimens were examined from the collections listed below. In addition, several colleagues sent records, images, and observations from personal collections (see acknowledgments). We checked European and Asian literature to ascertain if *Zanclognatha dentata* might represent an Old World species. Larval images (both film and digital) have been deposited at the University of Connecticut. We prepared 29 genitalic dissections of *Zanclognatha*: 12 of the *dentata*-*martha*-*protumnusalis* group (5 females, 7 males) and 17 dissections of five other species for comparison. In addition we examined 19 *Zanclognatha* genitalic preparations in Cornell University’s insect collection. Martin Honey and Don Lafontaine secured images of the types of *Zanclognatha protumnusalis* and *Zanclognatha minimalis* Grote in the British Museum.

AMNH	American Museum of Natural History, New York, New York, USA
            

BMNH	NaturalHistory Museum, London, England UK
            

CAES	Connecticut Agricultural Experiment Station, New Haven, Connecticut, USA
            

DH	Personal collection of Daniel Handfield, Saint-Mathieu de Beloeil, Québec, Canada
            

NYSM	New York State Museum, Albany, New York, USA
            

PMNH	Peabody Museum, Yale University, New Haven,Connecticut, USA
            

TLM	Personal collection of Timothy McCabe, Albany, New York, USA
            

UCMS	University of Connecticut, Storrs, Connecticut, USA
            

## Systematics

### Zanclognatha dentata

Wagner & McCabe sp. n.

urn:lsid:zoobank.org:act:6C1EC0E9-2F9A-4A6B-AC61-7F712FC6C9B8

http://species-id.net/wiki/Zanclognatha_dentata

Figs 1 [Fig F2] –9 

#### Type material.

**Figures 1–4.**
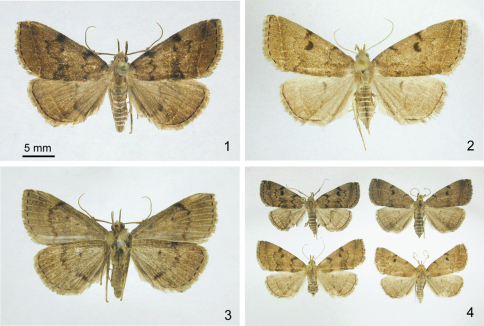
*Zanclognatha dentata* sp. n. **1** Holotype male, dorsal. CT: Tolland Co., Mansfield **2** Female, dorsal. CT: Windham Co., Hampton Reservoir **3** Holotype male, ventral **4** Variation. NJ: Atlantic Co., Egg Harbor Township (upper left); CT: Windham Co., Sterling (upper right); CT: Windham Co., Hampton Reservoir (lower left); MA: Franklin Co., Montague, Plains Road (lower right).

**Holotype** male (Fig. 1): USA, Connecticut, Tolland Co., Mansfield, Hunters Run, 41°46.18'N, 72°14.87' W, 4 July 2008, D. L. Wagner, mercury vapor light; DNA barcode voucher # CNCLEP 81920 (UCMS). **Paratypes** 54 males, 43 females. **Connecticut:** Litchfield County, Norfolk Great Mountain Forest, 13 July 1997, D. L. Wagner, (1 ♂) (UCMS) & 12 July 2008, D. L. Wagner, N. Proctor, A. Meleg (1 ♂) (UCMS); Middlesex County, East Haddam, Devil's Hopyard State Park, larvae 11 May 1994, 18 June, 1995, 20 June 1999, J. Fengler, J. Lozier, beaten from *Tsuga canadensis* (3 ♂) (CAES); New London County, Griswold, Hopeville Pond State Park, 9 July 1996, V. Giles (2 ♂) & 22 July 1997, F. Hohn (2 ♂) (UCMS); Tolland County, Mansfield, Hunters Run, 41°46'11"N, 72°14'52" W, 4–18 July 1997–2008, D. L. Wagner (3 ♂, 1 ♀) (UCMS); Windham County, Hampton, 2 July 1984, D. L. Wagner (1 ♂) (UCMS); Hampton Reservoir, NW of bog, 25–26 July 1996, D. L. Wagner & B. D. Williams (1 ♀) (UCMS); Catden Swamp, 25–26 July 1996, D. L. Wagner & B. D. Williams (1 ♀) (UCMS); Sterling Junction Rt. 14/14A, larva 30 June 2007, D. L. Wagner, beaten from and reared on *Lonicera merrowii*, emerged 18 July 2007, DLW Lot 2007F90.1 (1 ♀) (UCMS). **Maine:** Oxford County, Magalloway Plantation, State Route 16, 3 km NNE New Hampshire stateline, larva 5 June 1995, C. T. Maier, beaten from *Abies balsamea* (1 ♂) (CAES). **Massachusetts:** Franklin County, Montague Plain, 11 July 1991, D. L. Wagner, P. Z. Goldstein, & S. McKamey (1 ♂) (UCMS); Middlesex County, Concord, H.D. Thoreau's gravesite, 3–4 July 2009, D. L. Wagner (1 ♂) (UCMS). **Michigan:** Cheboygan Co., Pellston, Biological Douglas Lake, 7 July 2007, D. L. Wagner (1 ♂) (UCMS). **New Jersey:** Atlanta County, Egg Harbor Township, Absecon Creek, female 15 July 2002, D. F. Schweitzer, reared on dead oak leaves, emerged 13 Sept. 2002, DLW Lot 2002G117 (1 ♂) (UCMS); Atlantic County, Pomona, 6 July 1991, D. F. Schweitzer (1 ♂), gen. slide McCabe 2924 (TLM); Burlington County, Junction Route 563 & Wading River, 2 June 1999, D. L. Wagner, B. D. William, M. A. Volovski, & P. Mallard (1 ♂) (UCMS). **New Hampshire:** Coos County, Concord, 1 km NNE, North Concord, larva 4 June 1995, C. T. Maier, beaten from *Abies balsamea*, (1 ♂) (CAES); Pittsburg, Ildewide, west side of Second Connecticut Lake, larva 11 June 1996, C. T. Maier, beaten from *Abies balsamea*, JMF Lot 97–106 (1 ♂) (CAES). **North Carolina:** Haywood County, Cataloochee Campground, larva 10 June 2002, D. L. Wagner, beaten from *Hamamelis virginiana*, emerged 3 Aug. 2002, DLW Lot 2002E83 (1 ♀) (UCMS). **New York:** Albany Co., Pine Bush, 42°43.05' N, 73°52.16' W, 100 m, 2 July – 6 Aug. 1987–1997, T. McCabe (7 ♂, 3 ♀) (NYSM, TLM); Clinton Co., Gadway Barrens, 44°56.59'N 73°45.17'W, 180 m, 2 Aug. 1997, T. McCabe (2 ♀) (TLM); Essex Co., Lake Stevens, 44°22.58'N, 73°54.15'W, 1055 m, 6 June 1986, T. McCabe (1 ♂) (TLM); Franklin Co., Bloomingdale bog, 44°24.36'N, 74°07.24'W, 475 m, 2 Aug. 1997, T. McCabe (4 ♀), gen. slide McCabe 4188 & 4186 (NYSM, TLM); Hamilton Co., 6 mi. E. Indian L, 43°45.44' N,74°09.52' W, 555 m, 11 July–17 Aug. 1977–1980, T. McCabe (9 ♂, 6 ♀) (NYSM); Hamilton Co., 6 mi. E. Indian L, 43°45.44'N, 74°09.52'W, 555 m, 13 July 1977, T. McCabe (1 ♀, 1 ♂), gen. slide McCabe 1279 (NYSM); Orange Co., Cedar Pond bog, 44°56.59'N, 73°45.17'W, 180 m, 5 Aug. 2000, T. McCabe (1 ♂) (NYSM); Ulster Co., Lake Awosting, 41°42.43' N, 74°16.58' W, 550 m, 6 Aug. 1906, T. McCabe (1 ♀, 3 ♂) (NYSM); Ulster Co., Minnewaska St. Pk., 41°42.43'N, 74°16.58'W, 450 m, 6 Aug. 1906, T. McCabe (1 ♂) (NYSM). **Québec:** Val-Limoges, 200 km north of Ottawa, 46°39.8'N, 75°45.1'W, 14 July 2004, D. Handfield (2 ♀) (DH); La Présentation, 30 km east of Montréal, 45°41.3'N, 73°05.3'W, 30 June 2006, D. Handfield (2 ♂, 4 ♀) (DH); Sainte-Christine, 65 km east of Montréal, 45°39.0' N, 72°26.8' W, 3 July 2006, 20 July 2006, 28 July 2006, D. Handfield (2 ♂, 4 ♀) (DH); Villeroy, 175 km north-east of Montreal, 46°22.7' N, 71°49.9' W, 7 July 2006, D. Handfield (2 ♂) (DH); Saint-Narcisse, 200 km north-east of Montreal, 46°35.1' N, 73°11.9' W, 13 July 2006, D. Handfield (1 ♂, 2 ♀) (DH); Manseau, 150 km north-east of Montreal, 46°18.3'N, 72°00.7'W, 24 July 2006, D. Handfield (3 ♂, 9 ♀) (DH). **Vermont:** Essex County, Victory, Victory State Forest, 2.5 km SW Granby, larva 10 June 1997, C. T. Maier, on *Abies balsamea* (1 ♀) (CAES); Windham Co., Marlboro, Banks Road, 489 m, larva 15 June 1994, C. Lemmon, on *Abies balsamea*, Chris Maier Lot 94–89 (1 ♂, 1 ♀) (CAES).

#### Etymology.

The species name derives from the toothed antemedial and medial lines on the forewing.

#### Diagnosis (habitus).

Dark tooth-like spots along costa, marking beginning of antemedial and postmedial lines, distinguish Z. *dentata* from all but *Zanclognatha lituralis,* *Zanclognatha martha*, and some *Zanclognatha protumnusalis*. The presence of a third (subapical) costal spot, (where the subterminal line meets the costa), usually present in *Zanclognatha lituralis*, is absent in *Zanclognatha dentata*; the grayer ground color and uneven subterminal line also distinguish *Zanclognatha lituralis* from *Zanclognatha dentata*. *Zanclognatha martha* is distinguished from *Zanclognatha dentata* by its darker ground color, weakened subterminal line, darkened distal ¼ of forewing, and its larger size. The discal spot of *Zanclognatha dentata* tends to be larger, more vertically elongate, and the distal side is often more concave than that of *Zanclognatha protumnusalis* and others. The antemedial line of *Zanclognatha dentata* is more toothed (zigzagged) than that of most other similarly-sized, brown North American *Zanclognatha* (but see discussion). The postmedial line is often abruptly-angled outward over the radial veins in *Zanclognatha dentata*, whereas in *Zanclognatha protumnusalis* and *Zanclognatha martha*, this part of the postmedial tends to be more evenly rounded. In *Zanclognatha protumnusalis* the subterminal line is more likely to be outwardly edged with pale scales (in both wings) and *Zanclognatha protumnusalis* tends to have more tan in the ground color, thinner and crisper costal spots, and lacks the blurry patch of fuscous scales basad of the postmedial line, which extends from the inner margin to the cell, that is present in many *Zanclognatha dentata*. In most specimens of *Zanclognatha protumnusalis* the ground color of the hindwings tends to be noticeably paler than that of the forewings, especially through the radial area.

#### Diagnosis (genitalia).

**Figures 5–7. F2:**
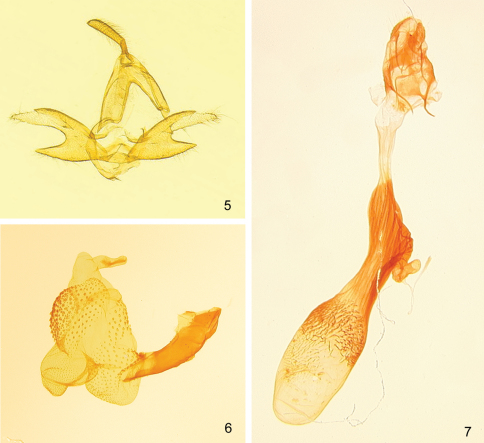
*Zanclognatha dentata* sp. n. genitalia. **5** male, NY: Hamilton Co., Indian Lake, McCabe diss. no. 1279. **6** aedeagus, same data. **7** female, NY: Franklin Co., Bloomingdale bog, McCabe diss. no. 4188.

(Figs 5–7). **Male genitalia** of *Zanclognatha dentata* differ significantly from those of *Zanclognatha lituralis* – most notably *Zanclognatha dentata* has the upper process of the valve adorned with a small tooth, which is only half as long as the width of the costal lobe, whereas *Zanclognatha lituralis* has a large tooth that is as long as the costal lobe is wide. *Zanclognatha lituralis* has a valve that resists spreading during genitalic preparation and becomes badly skewed if forced. *Zanclognatha martha* resembles *Zanclognatha dentata* but is larger. The spread valves of *Zanclognatha martha* expand to 3.0 mm whereas those of *Zanclognatha dentata* expand to 2.7 mm. *Zanclognatha dentata* male genitalia appear indistinguishable from those of Z. *protumnusalis* to our eye. **Female genitalia** have similar internal spinules in the corpus bursa in *Zanclognatha dentata*, *Zanclognatha lituralis*, *Zanclognatha martha*, and *Zanclognatha protumnusalis*, but these extend farther on one side of the bursa in *Zanclognatha protumnusalis* and *Zanclognatha dentata*. In our dissections, length of the female genitalia in *Zanclognatha dentata* is ≥ 6 mm in total length, whereas those of *Zanclognatha protumnusalis* length measure circa 5 mm.

#### Description.

Male. Forewing length: FWL 10.5–13 mm (n=30). **Head –** pale to deep brown with forward projecting tufts from vertex. Antenna with male androconial notch at 1/3. Labial palpus with third segment 1/2 length of second, with pale scales at apex; second segment with pale scales over mesal surface. **Thorax –** dorsum concolorous with head. Forewing subtriangular, pale to chocolate brown, and usually well marked. Antemedial line toothed or scalloped; discal spot usually well developed sometimes with distal side concave; postmedial line toothed, thickened where it joins costa; often with diffuse medial patch of dark scales from inner margin to cell; subterminal line straight, sparsely edged outwardly with pale scales. Hindwing brown with weak discal spot and variously-developed postmedial and subterminal lines; the latter generally poorly differentiated to absent. If outwardly edged with pale scales, usually only over anal and cubital areas of wing. Underside of both wings usually with discal spot and well-expressed postmedial line. Procoxa elongate with yellow androconia. Profemur with (concealed) yellow hair pencil from distal end and fan of dark androconial scales from proximal end—both of which usually folded and covered by broad hood of chocolate colored scales from protibiae. Mesothoracic and metathoracic tibiae and tarsomeres lightened apically, appearing banded in dark individuals. **Abdomen –** Tan to brown with distal edge of each segment pale: abdomen appearing banded in well-marked individuals. **Male genitalia** (Figs 5, 6). **Valves** (Fig. 5) **–** Nearly symmetrical; uncus distally expanded compressed laterally, terminating in minute tooth; tegumen as long as valve; valve divided into two lobes for half its length; costal (upper) lobe with short mesal tooth halfway along length; costal lobe terminating in irregular apex crowned with setae, with apices of left and right valves differing in detail; lower lobe unadorned. **Aedeagus** (Fig. 6) **–** everted vesica covered with spinules; simple basal lobe; slightly curved mesal lobe; large distal lobe supports very small bulge without spicules. **Female genitalia** (Fig. 7) **–** Papillae anales unmodified, short; anterior and posterior apophyses subequal in length; distal half of ductus bursae lightly sclerotized, then heavily sclerotized and ribbed to beyond ductus seminalis; ductus seminalis short and twisted; caudal half of corpus bursae with relatively long, curved, internal spinules; spinules extend past middle of corpus bursae on side opposite ductus seminalis.

#### Remarks.

Dark, boldly-marked individuals are commonly encountered southward. In some, the medial patch of dark scales extends across the wing. Adult phenotypes overlap with those of *Zanclognatha protumnusalis* to the extent that we cannot reliably assign about 15% of light-trapped adults to one species or the other. No diagnostic genitalic characters are known for either sex. COI barcodes for those individuals that we could reliably identify were diagnostic (see below). The holotype was submitted to BOLD for COI barcoding (CNCLEP 81920) and its sequence will be submitted to GenBank. Larval features are also diagnostic (for both species).

#### Distribution.

So far as known, Ontario to Nova Scotia southward through the Great Lake states and in the Appalachians to northern Georgia. One moth from a sandhills area in central South Carolina appears to represent *Zanclognatha dentata*, but we excludethe moth from the type series.

#### Biology.

Adults have been taken at lights and sugar bait from a broad range of habitats that includes bogs, swamps, marshes, Atlantic white cedar swamps, swales, and other wetlands, mesic hardwood and Appalachian cove forests, a variety of boreal (conifer) forest types, and pitch pine/scrub oak barrens. The species is essentially univoltine throughout most of its range with a single mid-summer flight from the end of June through early August, with more than 80% of the adults from New Jersey northward taken in July. Records from early September in western North Carolina and northern Georgia by James Adams (pers. comm.) are indicative of a small second brood, as also occurs in *Zanclognatha protumnusalis* and others (Wagner et al. 2011).

Chris Maier, Jeff Fengler, and Carol Lemmon, made numerous collections of *Zanclognatha dentata* during their survey of conifer-feeding caterpillars of the Northeastern United States (Maier et al. 2004). Nine of their larval collections were reared to the adult stage; larval images for four additional collections are assignable to *Zanclognatha dentata*. Their host records include: *Abies balsamea* (L.) Mill. (n=7), *Tsuga canadensis* (L.) Carrière (n=3), and *Pseudotsuga menziesii* (Mirb.) Franco. We have taken singleton larvae in beating sheet samples on three occasions: from *Hamamelis virginiana* L., *Lonicera morrowii* A. Gray, and a third, unrecorded host. All of the above were taken in May and June as penultimate or final instars. Although *Zanclognatha* species are generally regarded to be litter dwellers (Crumb 1956; Hohn and Wagner 2000; Wagner 2005), at least three other members of the genus (in addition to *Zanclognatha dentata*) are known to feed above the ground: *Zanclognatha theralis* (Walker, 1859) in *Usnea* lichens (Sigal 1984); *Zanclognatha protumnusalis* in fir, spruce, pines, and other conifers (Prentice 1962 and reared specimens in the PMNH); and *Zanclognatha martha* a hard pine associate (Wagner et al. 2011). We also have taken *Zanclognatha cruralis* (Guenée, 1854) and related species on occasion while beating low woody and herbaceous vegetation in forests, but mostly in the fall, before leaf fall, and not in the spring as has been the case with the four *Zanclognatha* listed above. Dale Schweitzer and DLW reared an ex ova cohort of *Zanclognatha dentata* through to maturity on a diet of dead oak leaves (DLW Lot 2002G117).

**Figures 8–11.**
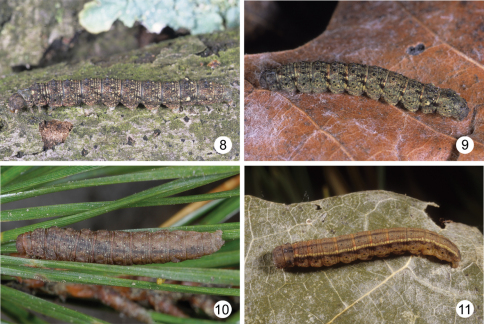
Last instar *Zanclognatha*. **8** *Zanclognatha dentata* sp. n.: NC: Haywood Co., Great Smoky Mountain National Park, Catalochee Campground, DLW Lot 2002E83, larva and photo 10 June 2002. **9** *Zanclognatha dentata* **sp. n.:** NJ: Atlantic Co., Egg Harbor Township, Abescon Creek, female (mother) 15 July 2002; photo: 24 August 2002, DLW Lot 2002G117 **10** *Zanclognatha martha*. NY: Clinton Co., Clintonville, Dry Bridge Road, N44°28'14", W73°36'15", 660ft, larva 25 June, 2008, on *Pinus rigida*, DLW Lot 2008F200 **11** *Zanclognatha protumnusalis*. CT: New London Co., Griswold, Hopeville Pond State Park, female 10 August, 1997, image November 1997, Fred Hohn lot number F263.

## Larval Identification

The caterpillar is mottled in brown, red, and yellow with a conspicuous pale subdorsal spot on A7 (Figs 8–9). Below we expand Crumb’s (1956) key to *Zanclognatha* larvae by including four new taxa: *Zanclognatha dentata*, *Zanclognatha martha*, *Zanclognatha protumnusalis*, and *Zanclognatha marcidilinea* (Grote, 1872). Given the confusing taxonomy of the genus (e.g., the possibility of misidentifications, especially in historic material), our small sample sizes, intraspecific (including ontogenetic) variability in larval phenotypes, our key should be considered preliminary. Development of the middorsal stripe, emphasized by Crumb (1956) and below, is variable. For example, our example of *Zanclognatha marcidilinea* had only a weak line, best expressed over the thoracic segments; conversely *Zanclognatha cruralis* sometime has the remnants of a middorsal line (broken between segments). Last instars of all ten species are figured in Wagner et al. (2011).

### Preliminary key to *Zanclognatha* larvae

**Table:** 

1	Ground color smoky to charcoal; body somewhat corrugated (constricted between segments); red reticulations inconspicuous; larva arboreal on pitch and perhaps other hard pines	*Zanclognatha martha*
–	Ground color with variously developed red reticulations at least laterally and otherwise not as above	2
2	Middorsal stripe usually present over abdomen; ground colors various	3
–	Middorsal stripe usually absent over abdomen; ground color with conspicuous red reticulations	8
3	Middorsal stripe fuscous (dark), contrasting with adjacent dorsal coloration over abdomen	4
–	Middorsal stripe reddish, weakly contrasting with adjacent dorsal coloration, evident mostly over thorax	6
4	Dorsum with red mottling that often joins into lines along inner side of creamy subdorsal stripe	*Zanclognatha protumnusalis*
–	Not as above; mottling not coalescing into red lines and without a creamy subdorsal stripe; dorsal area reticulated in gray, brown, red, and yellow	5
5	Ground color smoky;dorsal pinacula over abdomen slightly enlarged, diameter > than height of spiracle on A8; diffuse, dark oblique lines extending forward and upward from spiracle, especially on A2–A6 (A7)	*Zanclognatha pedipilalis*
–	Ground color reddish; dorsal pinacula over abdomen slightly enlarged, diameter < than height of spiracle on A8; without set of well-developed, dark oblique lines extending forward and upward from spiracle, especially on A2–A6	*Zanclognatha laevigata*
6	A7 with pale supraspiracular spot	7
–	A7 without pale supraspiracular spot; often with oblique, arching yellow line, between dorsal pinacula on A1–A7	*Zanclognatha marcidilinea*
7	Dorsal abdominal pinacula wartlike, conspicuously wider and higher than those over thoracic segment, diameters > height of A8 spiracle; pale spot on A7 from (raised) supraspiracular swelling (bearing SD1)	*Zanclognatha dentata* sp. n.
–	Dorsal abdominal pinacula flatter, diameters similar to those over thoracic segments, < height of A8 spiracle; subdorsal area of A7 not conspicuously swollen	*Zanclognatha lituralis*
8	Dorsal abdominal pinacula small; diameters equal to those over thoracic segment and diameter < height of A8 spiracle	*Zanclognatha theralis*
–	Dorsal abdominal pinacula distinctly larger than those over thorax, diameters ≥ height of A8 spiracle	9
9	Dorsal abdominal pinacula enlarged but diameter of D1 pinacula < 1/4 distance that separates them across midline; D1 pinaculum < 2 × diameter of D2 pinaculum	10
–	Dorsal abdominal pinacula larger with diameter of D1 pinacula about 1/3 distance of that separating them across midline; D1 pinaculum > 2 × that of D2 pinaculum	*Zanclognatha jaccusalis* (= *Zanclognatha ochreipennis* of Crumb 1956)
10	D1 and D2 pinacula over abdominal segments subequal; southern US	*Zanclognatha obscuripennis*
–	Diameters of D1 pinacula larger than those of D2 over abdominal segments; widespread (common)	*Zanclognatha cruralis*

## Discussion

*Zanclognatha dentata* is widespread in Northeastern North America. In most collections it is intermixed with *Zanclognatha protumnusalis*. Less often specimens are sorted with those of *Zanclognatha lituralis* and *Zanclognatha martha*. Surprisingly, given the number of wing scaling characters that distinguish *Zanclognatha dentata* from other *Zanclognatha*, we did not find male or female genitalic differences that would reliably separate the new species from *Zanclognatha protumnusalis*. As noted above, genitalia are somewhat generalized across the genus (see Forbes 1954: 389 for illustrations of the valve and aedeagus for seven species). As is suggested by the larval key, all of the aforementioned species of *Zanclognatha*, and others, can be distinguished based on larval characters (see also Crumb 1956).

Daniel Handfield had individuals of both *Zanclognatha protumnusalis* (n = 37) and *Zanclognatha dentata* (n = 31) from Quebec sequenced as part of the Bar Codes of Life Data System (http://www. barcodinglife.org/views/login.php). His *Zanclognatha dentata* (included as paratypes here) clustered in two haplotype groups, remote from those of *Zanclognatha protumnusalis* which clustered in a single group that included several haplotypes. In a larger *Zanclognatha* data set (n=251) that includes barcodes of all named eastern species, most *Zanclognatha protumnusalis* clustered with *Zanclognatha cruralis*, *Zanclognatha jaccusalis* (Walker, 1859) and *Zanclognatha obscuripennis* (Grote, 1872), whereas *Zanclognatha dentata* haplotypes clustered with *Zanclognatha martha*, *Zanclognatha theralis* (Walker, 1859) (in part) and *Zanclognatha atrilineella* (Grote, 1873) (Don Lafontaine in litt.). That the two species were not each other’s sisters is hardly surprising given the differences in larval phenotypes of the two: compare figures 8 and 9 with 11 and differences enumerated in the larval key.

The type of *Zanclognatha minimalis* Grote, 1878, currently regarded as a synonym of *Zanclognatha protumnusalis* (e.g., Franclemont and Todd 1983) (BMNH), is somewhat intermediate in character between *Zanclognatha dentata* and *Zanclognatha protumnusalis*. On the whole *Zanclognatha minimalis* aligns best with the latter, e.g., hindwings of *Zanclognatha minimalis* are too pale to fit comfortably within *Zanclognatha dentata* and the discal spot is small, round and almost free of dark scales. However, two features of the type give us pause: (1) the postmedial line is strongly expressed to the inner margin (it is nearly always vague below the cell in *Zanclognatha protumnusalis*). (2) Likewise the antemedial line is toothed and well expressed to the inner margin, and thus more reminiscent of *Zanclognatha dentata*.

The New York State Museum has no historical records of *Zanclognatha dentata*, and TLM did not find this species in Ithaca when he collected there at both light and bait from 1974–1975. The earliest specimens that we have been able to locate are a series in the American Museum of Natural History from Ocean County, New Jersey taken in 1936. And while the recency of discovery of what is now a widespread and common moth would suggest the species could be an introduction, its presence in northern bogs in Quebec, barrens in New Jersey (in the 1930s), and cove forests in the Great Smokies Mountains are indicative that *Zanclognatha dentata* is a native that escaped the attentions of early lepidopterists.

*Zanclognatha* is in need of revisionary study. The number of valid species in the *gypsalis-theralis-deceptricalis-inconspiculis* complex is unknown. *Zanclognatha lituralis* consists of at least two valid species (Rings et al. 1992). We are uncertain if what is being called *Zanclognatha martha* in the Great Lakes Region is conspecific with the pitch pine-feeding populations of the Eastern seaboard states. Likewise, *Zanclognatha protumnusalis* (even with *Zanclognatha dentata* removed) may not be a single entity, e.g., specimens from along the Gulf Coast may be nominally distinct. COI barcodes and other (nuclear) molecular data will be needed in many cases, as both genitalia and wing patterns are often of limited utility in the genus. Larvae of *Zanclognatha* are diverse in character given the modest differences in adult features (*Zanclognatha dentata* was initially recognized as a distinct entity based on its larva). We encourage others to rear ex ova broods and to preserve and photograph larvae as circumstances permit—most *Zanclognatha* can be reared on dead oak, cherry, and birch leaves.

## Supplementary Material

XML Treatment for Zanclognatha dentata
